# A novel approach for modelling vegetation distributions and analysing vegetation sensitivity through trait-climate relationships in China

**DOI:** 10.1038/srep24110

**Published:** 2016-04-07

**Authors:** Yanzheng Yang, Qiuan Zhu, Changhui Peng, Han Wang, Wei Xue, Guanghui Lin, Zhongming Wen, Jie Chang, Meng Wang, Guobin Liu, Shiqing Li

**Affiliations:** 1 State Key Laboratory of Soil Erosion and Dryland Farming on the Loess Plateau, Northwest A&F University, Yangling, Shaanxi, China; 2 Department of Biology Sciences, Institute of Environment Sciences, University of Quebec at Montreal, Montreal, Canada; 3 Center for Earth System Science, Tsinghua University, Beijing, 100084, China; 4 College of Life Sciences, Zhejiang University, Hangzhou, 310058, China

## Abstract

Increasing evidence indicates that current dynamic global vegetation models (DGVMs) have suffered from insufficient realism and are difficult to improve, particularly because they are built on plant functional type (PFT) schemes. Therefore, new approaches, such as plant trait-based methods, are urgently needed to replace PFT schemes when predicting the distribution of vegetation and investigating vegetation sensitivity. As an important direction towards constructing next-generation DGVMs based on plant functional traits, we propose a novel approach for modelling vegetation distributions and analysing vegetation sensitivity through trait-climate relationships in China. The results demonstrated that a Gaussian mixture model (GMM) trained with a LMA-N_mass_-LAI data combination yielded an accuracy of 72.82% in simulating vegetation distribution, providing more detailed parameter information regarding community structures and ecosystem functions. The new approach also performed well in analyses of vegetation sensitivity to different climatic scenarios. Although the trait-climate relationship is not the only candidate useful for predicting vegetation distributions and analysing climatic sensitivity, it sheds new light on the development of next-generation trait-based DGVMs.

Terrestrial vegetation plays a crucial role in land surface processes, carbon and nitrogen cycles, and water and heat fluxes via biogeochemical processes^1^. Dynamic global vegetation models (DGVMs) are state-of-the-art tools used to describe the structures and functions of the terrestrial biosphere as well as water and energy cycling on the land’s surface^2^,^3^. In the past decade, plant functional types (PFTs) have been widely adopted in most DGVMs to evaluate the response of vegetation to climate change^4^,^5^,^6^. PFTs are defined as groups of plant species that either exhibit similar responses to environmental conditions or display similar ecological structures and functions^7^,^8^. PFT schemes have performed well in simulations of global vegetation dynamics under current climate change conditions and have successfully reconstructed palaeo-vegetation patterns^2^,^9^,^10^.

DGVMs simulate and predict vegetation distributions based on the assumption that the vegetation distribution is directly controlled by climatic conditions^4^,^11^. It has generally been assumed that PFT schemes are fully capable of simulating the dynamic process involved in the response to climate change in DGVMs; however, a PFT scheme is not sufficient for simulating ecological processes. In most cases, the PFTs defined in DGVMs have been pre-described with fixed traits and have been assumed to respond to physical and biotic factors in a static manner; however, these traits exhibit great variability in the real world^12^,^13^. In addition, clear divergence from real-world conditions arises when vegetation types are grouped into PFTs based on mean trait values, causing the variability among vegetation types to be lost^12^. Moreover, future climates may have no analogue in present climate conditions, leading to a lack of corresponding PFTs for future climate scenarios^14^,^15^.

Plant functional traits (FTs) are observed or measurable characteristics of plants that are assumed to reflect evolutionary responses to external environmental conditions^16^. FTs are defined as morphological, physiological and phenological traits that impact individual fitness indirectly via their effects on growth, reproduction and survival^17^. FTs vary consistently along environmental gradients and can to some extent be considered “response traits”, resulting from the filtering effect of climatic, disturbance and abiotic conditions^18^,^19^,^20^. Current DGVMs rely on earlier classifications, such as that of Box^21^, which is a simple scheme with explicit bioclimatic limits and PFTs that are not fully characterized in terms of the traits they represent^22^. Therefore, treating plant species as a set of FTs to replace fixed PFTs would greatly increase our capacity to predict an ecosystem’s structure and function^20^,^23^. FT-based theories are more closely related to evolutionary selection mechanisms^14^ and are regarded as a priority in the new generation of DGVM development^14^,^24^,^25^,^26^,^27^. Additionally, under certain scenarios of future climatic or land-use conditions, trait-climate approaches could help us to better understand ecosystem structures and processes^23^,^28^.

Vegetation change can affect the climate via feedbacks altering the surface albedo, surface energy fluxes, and hydrological cycling^29^, thereby influencing the productivity and budget patterns of an ecosystem. Evaluating and predicting the distribution of vegetation types is one of the principle aims of DGVMs. Recently, Van Bodegom *et al*.^30^ provided proof of principal with respect to the development of a trait-based approach for predicting the global vegetation distribution using three selected traits and Gaussian mixture density functions, paving the way for constructing a new generation of trait-based global vegetation models. Unfortunately, this fully trait-based vegetation approach correctly predicted only 42% of the observed vegetation distribution.

China is a country with abundant vegetation biomes extending across several climate zones, from tropical to boreal, and it exhibits the world’s largest and highest plateau^31^. Annual average temperatures range from −21.0 °C to 26.0 °C in China and increase from north to south, while precipitation ranges from 0 to 2250 mm and decreases from southeast to northwest (Fig. S1). The complexity and diversity of the country’s vegetation makes China an ideal test bed for vegetation modelling. Many previous studies have attempted to model vegetation distributions using empirical vegetation-climate relationships or PFT-climate schemes^32^,^33^,^34^,^35^, but all of these studies had difficulty simulating the vegetation distribution in China because a small number of PFTs (commonly fewer than 12) cannot fully represent the behaviours of all vegetation types. Therefore, there is an urgent need to develop methods (such as trait-based approaches) to replace PFT-climate schemes for predicting vegetation distributions under different climatic conditions in China. In this study, we propose a new trait-based framework for improving the PFT climate scheme in DGVMs based on findings from previously published studies^14^,^27^,^36^. The usefulness of vegetation models depends strongly on their ability to correctly predict the vegetation distribution under different climatic scenarios. Thus, the major objectives of this study were to (1) develop a new framework for modelling vegetation distributions based on trait-climate relationships, (2) simulate vegetation distributions across China, and (3) investigate the response of vegetation ecosystems to a changing climate through sensitivity analysis.

## Results

### Trait-climate relationships

**Figure 1:**
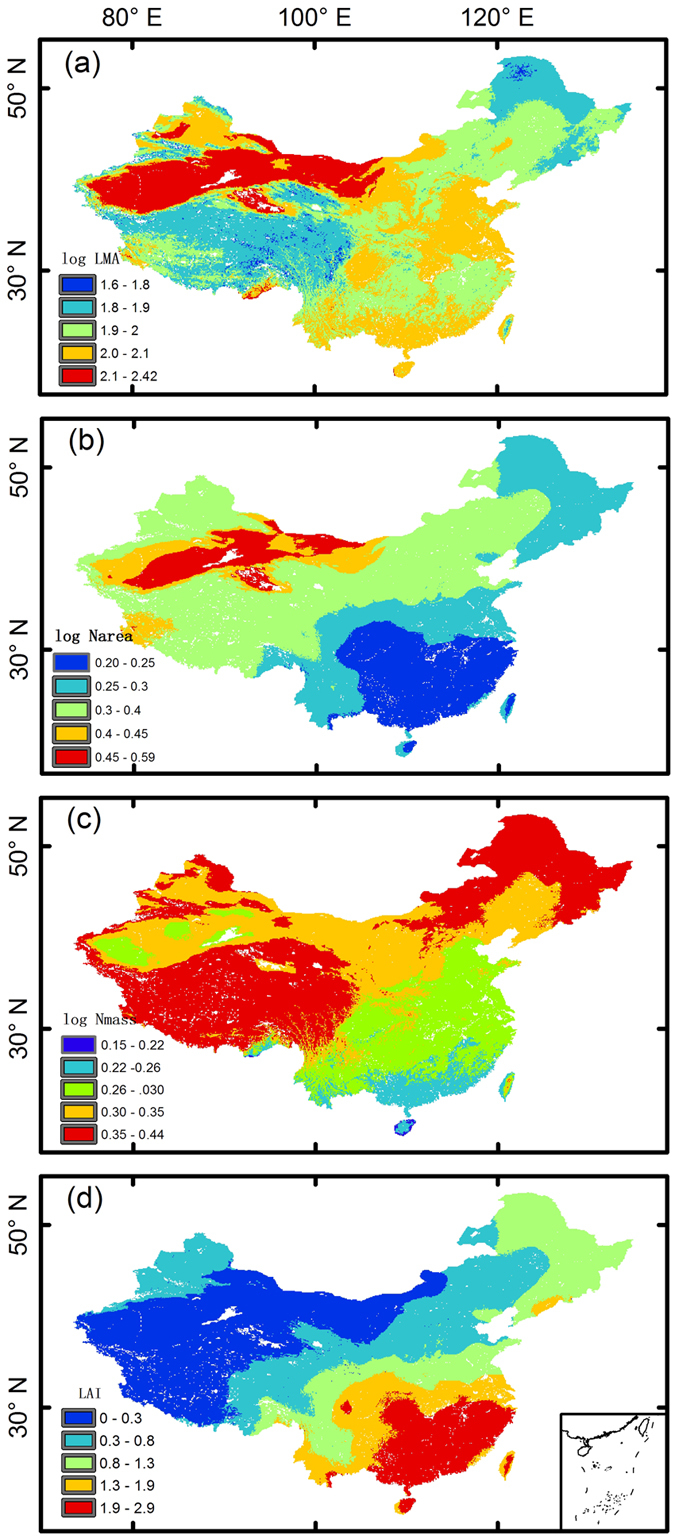
Log (LMA), Log (N_area_), Log (N_mass_) and LAI patterns predicted based on trait-climate relationships. LMA = leaf mass per area; N_area_ = area-based leaf nitrogen; N_mass_ = mass-based leaf nitrogen. The maps were generated using ArcGIS 10.2, http://www.esri.com/.

Global linear regressions of LMA-climate and N_mass_-climate data have been established^37^,^38^, and these regressions were updated after adding traits specific for China (Table 1). Three plant functional traits and MAP were approximately log-normally distributed; thus, they were log10-transformed according to the method described by Wright *et al*.^39^ before analysis. In general, vegetation distributions are sensitive to trait-climate interactions. LMA measures the leaf dry-mass investment per unit of light-intercepting leaf area and is the inverse of the specific leaf area (SLA). LMA increased with increasing temperature and exhibited a tendency towards higher values at lower levels of precipitation. Species with high LMA commonly exhibit thick leaf blade, dense tissue or both^40^, showing adaptation to arid environments. The leaf nitrogen concentration (both mass-based and area-based) is integral to the proteins involved in the Rubisco complex^40^, and is essentially influenced by temperature. The potential ways in which such an influence could occur are complex among different functional types and different regions. Leaf N_mass_ decreases with increasing temperature, indicating that alpine and arctic plant species display a high leaf N_mass_ compared with plants in warmer areas. N_area_ is defined as N_mass_ × LMA, representing adaptation to drought and water conservation^26^. As expected, plants in dry areas exhibited higher N_area_. In agreement with previous publications^39^, N_area_ increased as a function of increasing irradiance and decreasing annual precipitation. As a structural trait of plant communities, the leaf area index (LAI) is an indicator of canopy cover and annual leaf turnover (only for deciduous trees), and it is greatly influenced by the mean annual precipitation (MAP). The observed LAI was consistent with the distribution of MAP to some extent and showed a slightly negative relationship with RAD (Fig. 1 and Fig. S1). The constructed trait-climate relationships were applied in predicting trait distributions under different climatic conditions.

The spatial patterns of LMA, N_area_, N_mass_ and LAI (Fig. 1) were predicted using the trait-climate relationships provided in Table 1. Log (LMA) was affected by temperature and precipitation, which were high in temperate deserts and low on the Qinghai- Tibet Plateau. Log (N_area_) decreased from southeast to northwest in China, with the desert exhibiting the lowest value. Log (N_mass_) was controlled by temperature, presenting a positive relationship with temperature. LAI was affected by both MAP and RAD, exhibiting high values in the southeast and low values in high RAD areas.

### Classification results using GMM methods

**Figure 2:**
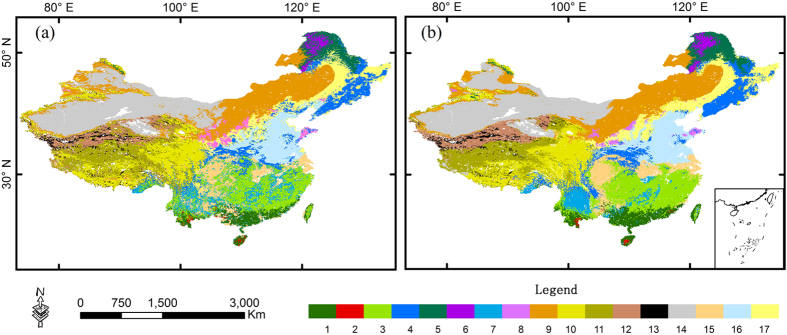
Natural vegetation map (**a**) and classification results obtained using the GMM classifier (**b**). a, Tropical rain forests (TrF); b, Tropical monsoon forests (TmF); c, Subtropical forest complexes (SuF); d, Temperate deciduous forest complexes (TDF); e, Boreal evergreen needle-leaf forests (BEF); f, Boreal deciduous broadleaf forests (BDF); g, Evergreen shrublands (ES); h, Deciduous shrublands (DS); i, Temperate steppe (TS); j, Alpine meadow (AM); k, Alpine steppe (AS); l, Alpine desert (AD); m, Tundra (Tu); n, Desert (De); o, Subtropical crops (SC); p, Temperate crops (two crops per year) (TCT); q, temperate crops (one crop per year) (TCO). The maps were generated with ArcGIS 10.2, http://www.esri.com/.

**Figure 3:**
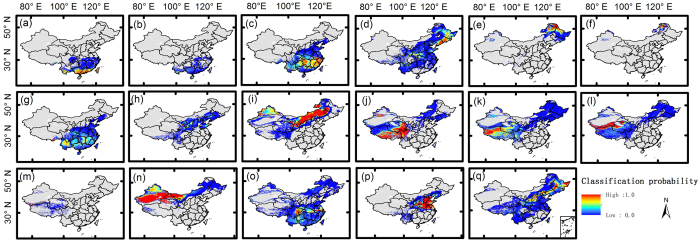
Classification probability of each plant functional type. From top left to bottom right, vegetation “a” to “q”; the order is consistent with that in Fig. 2. The maps were generated with ArcGIS 10.2, http://www.esri.com/.

We tested all of the models listed in Table 2 and compared the results. The results demonstrated that (1) in all 11 models, the GMM trained by the N_mass_-N_area_-LMA combination exhibited the highest accuracy (overall accuracy = 73.46%; kappa coefficient = 0.85); and (2) the optimal number of traits was three, with this model showing higher accuracy than the 2-trait and 4-trait combinations. N_area_ and N_mass_ can be interconverted via LMA (i.e., N_area_ = N_mass_ × LMA); thus, the N_mass_-N_area_-LMA combination shows limited predictive ability when it is integrated into DGVMs. The LMA-N_mass_-LAI combination exhibited similar accuracy (overall accuracy = 72.82%; kappa coefficient = 0.85) (Fig. 2) and could provide more parametric information about community structure and ecosystem function. Therefore, the LMA-N_mass_-LAI combination was applied in training the GMM for the analysis of vegetation-climate relationships and the response of vegetation patterns to climate change. The probability distribution map (Fig. 3) was consistent with the natural vegetation map, indicating that the trained GMM was sufficiently accurate (Table S2) for application in modelling vegetation distributions in China based on FTs.

**Figure 4:**
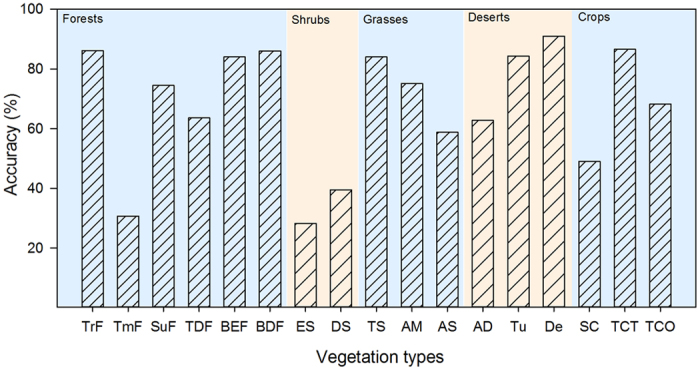
Classification accuracy of each vegetation type.

At the biome level, the accuracy of 13 vegetation types exceeded 60%, exhibiting satisfactory classification results (Fig. 4). In the GMM classification, deserts presented the highest average accuracy, of 79.36%, followed by grasses (72.64%), forests (69.18%), crops (67.95%) and shrubs (33.91%). In traditional DGVMs, such as BIOME4, the highest average accuracy is observed for forests (60.45%), followed by tundra and desert (49.9% on average) and then grasses (32.5%)^41^. Our results improve upon previous work regarding biome accuracy. Compared with a fully trait-based method, this method improves the predicted accuracy from 42% to 73% and overcomes the data limitations of a fully trait-based method to a certain extent.

### Vegetation patterns under six important scenarios

**Figure 5:**
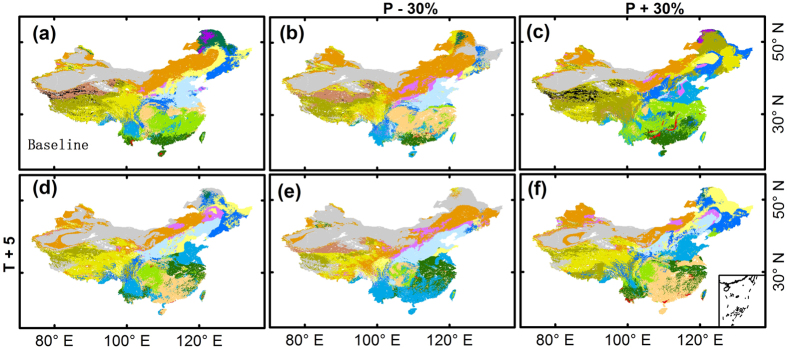
Projected vegetation patterns under six climatic sensitivity scenarios (increasing temperature by 5K; changing precipitation by ±30%). The legend is the same as for Fig. 2. The vegetation baseline map was generated using the average meteorological data between 1987 and 2013. The maps were generated with ArcGIS 10.2, http://www.esri.com/.

We selected six typical climate scenarios to describe the vegetation response to climate change. The results regarding vegetation patterns under the six climate scenarios are shown in Fig. 5. A 30% decrease in precipitation reduces the area occupied by forests and expands grassland areas (Fig. 5b). The boundaries of the temperate steppe shift eastward, and subtropical crops occupy most areas of the subtropical region, whereas two-crop-per-year temperate crops remain nearly unchanged. Tropical forests also remain unchanged compared with the baseline map (Fig. 5a). Desert and alpine desert regions show little difference under this climate scenario. Simultaneously, the alpine steppe shifts southward and occupies a large area of the Qinghai-Tibet Plateau.

Increasing precipitation by 30% expands forested lands and shrinks grasslands (Fig. 5c). In tropical regions, tropical forests shift northward. The subtropical region is predominantly covered by subtropical forest complexes. Evergreen shrublands distributed on the Yunnan-Guizhou Plateau are replaced by subtropical forests. Temperate forests expand to cover a larger area in temperate regions. The temperate steppe and alpine desert shrink, and the desert located in the north of Xinjiang is replaced by a temperate steppe. Boreal forests shrink marginally compared with the baseline map. Additionally, the alpine steppe occupies most of the Qinghai-Tibet Plateau.

Increasing the temperature by 5 K shifts the predicted boundaries of most vegetation types northward and westward (Fig. 5d). Tropical forests shift northward, and evergreen shrublands expand to a larger area than on the baseline map. The North China Plain is also partially occupied by subtropical shrublands. The temperate forests shift northward. Two-crop-per-year temperate crops shift northward and occupy part of northeast China. The temperate steppe shrinks compared with its baseline area. The alpine desert also shrinks, and the tundra disappears from the Qinghai-Tibet Plateau.

Under the climate scenario of a 30% decrease in precipitation and a 5 K increase in temperature by, the temperate forest practically disappears and is only distributed in northeastern China (Fig. 5e). The temperate steppe also shrinks. In tropical and subtropical regions, evergreen shrublands occupy most of the area and they expand to part of northern China due to their adaptations to hot temperatures and low precipitation. Subtropical forest complexes shrink. The boundaries of tropical rain forests and tropical monsoon forests shift northward, with these forests being distributed along the Yangtze River. Deserts expand to a larger area compared with the baseline map. Vegetation on the Qinghai-Tibet Plateau is sensitive to this climate scenario. Boreal forests disappear in China due to their adaptation to cold scenarios.

Under the climate scenario of a 30% increase in precipitation and a 5 K increase in temperature, the boundaries of vegetation communities shift northward and westward (Fig. 5f). Tropical forests shift northward and are distributed along the Yangtze River. The Yunnan-Guizhou Plateau is also occupied by tropical forest complexes. In subtropical regions, subtropical crops are distributed throughout a larger area, without consideration of topography. In temperate regions, temperate forest complexes appear to the north of the Loess Plateau, and the boundaries of temperate crops (i.e., both two-crop–per-year and one-crop–per-year systems) shift northward. Boreal deciduous forests disappear. Deserts shrink compared with the baseline map. The temperate steppe shifts westward and shows a slightly decrease in area. On the Qinghai-Tibet Plateau, the boundaries of the alpine meadow shift northward, occupying most of this region. As expected, alpine deserts and tundra disappear or decline under this climate scenario.

### Sensitivity analysis

**Figure 6:**
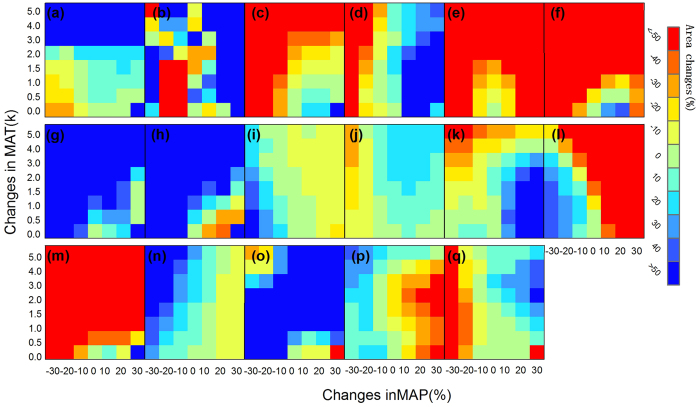
Sensitivity analysis of the distribution areas of 17 vegetation biomes under different climate scenarios. The seventeen frames from the upper left to lower right correspond to the vegetation types from “a” to “q”; the order is consistent with that in Fig. 2. MAT = mean annual temperature; MAP = mean annual precipitation. The maps were generated with ArcGIS 10.2, http://www.esri.com/.

An increasing temperature shifts most forest boundaries northward and westward and expands the evergreen shrublands habitat to a larger area compared with the baseline (Fig. 5d–f). With the exception of tropical forests and subtropical forest complexes, forest biomes exhibit a decreasing trend as the temperature increases (Fig. 6c–f). Evergreen shrublands and deciduous shrublands are sensitive to increasing temperature (Fig. 6g,h) and expand to a larger region when the temperature increases compared with the baseline (Fig. 5a). Alpine steppe and alpine desert regions exhibit a decreasing trend with increasing temperature, whereas alpine meadows increase, indicating that the Qinghai-Tibet Plateau region is sensitive to a changing temperature. As expected, the area of temperate desert increases when the temperature rises, and precipitation decreases or remains unchanged (Fig. 6n). Subtropical crops increase initially and then decrease as the temperature increases (Fig. 6o). By contrast, two-crop-per-year temperate crops first decrease and then increase as the temperature rises (Fig. 6p). One-crop-per-year temperate crops show only small changes under increasing climate conditions (Fig. 6q).

Increasing precipitation expands most forest biomes to a larger area than the baseline. An exception to this relationship is that boreal forests exhibit only small changes, first increasing in area and then decreasing (Fig. 6e,f). Evergreen shrublands and deciduous shrublands display a decreasing trend when precipitation increases (Fig. 6g,h). The temperate steppe shrinks and is replaced by one-crop-per-year temperate crops (Fig. 5c,f). On the Qinghai-Tibet Plateau, the alpine meadow shrinks when precipitation decreases; however, it increases slightly as precipitation increases. Unexpectedly, the alpine steppe increases when precipitation increases. As expected, both alpine desert and tundra decrease as precipitation increases, and temperate deserts present a similar response to precipitation. Under unchanged temperature conditions, subtropical crops increase as precipitation decreases and decrease as precipitation increases; however, these crops exhibit positive behaviour when the temperature increase is greater than 3 K. Two-crop-per-year temperate crops decrease as precipitation increases; however, one-crop-per-year temperate crops exhibit a positive relationship with increasing temperature.

## Discussion

This study applied trait-climate relationships to classify vegetation with the aid of a GMM classifier for the first time in China. Compared with the natural vegetation distribution, the kappa coefficient obtained in this study (0.85) is broadly consistent with the results of Yuan *et al*.^34^ and Wang *et al*.^35^, who obtained kappa coefficients of 0.76 and 0.75, respectively. Trait-climate relationships enable detailed information about agricultural vegetation and the vegetation of the Qinghai-Tibet Plateau to be presented. Human activities generally make it difficult to simulate agricultural vegetation in DGVMs. However, this study incorporated three types of agricultural vegetation in the simulations. Although topographical factors and human activities were not considered, this study revealed the most suitable growth area and its response to a changing climate. The Qinghai-Tibet Plateau is a region of interest due to its unique location, elevation and climate. In the present study, we divided the vegetation in this region into three types according to different climatic conditions, which allowed greater sensitivity and detail to be obtained regarding the response of the alpine vegetation to climate change, and the results supported the hypothesis that this region is vulnerable to climate change.

GMMs have been successfully accepted and applied for the prediction of global vegetation distributions through an FT-based approach^27^,^30^. A fully trait-based vegetation map predicted 42% of the observed vegetation distribution correctly^30^. In the present study, we improved the prediction accuracy to 73% based on an FT model. The difference between the two studies lies in the training dataset used for the GMM classifier. Calibration traits and vegetation types were used to train GMMs in a study by Van Bodegom *et al*.^30^; by contrast, calibrated vegetation types and predicted traits were used as training samples in our study. This method can overcome insufficient trait data and effectively improve prediction accuracy. Moreover, in the study by Van Bodegom *et al*.^30^, only 9 vegetation types were considered for global vegetation, which may be insufficient to capture the complexity and diversity of Chinese vegetation and appears to be too coarse for modelling the spatial distribution of Chinese vegetation at regional or national scales^10^.

The regression coefficients of LMA-climate and N_mass_-climate relationships were still low in this study, showing little improvement compared with previous studies^38^,^39^,^40^. More effective trait-climate relationships should be developed in the future. CO_2_ has direct physiological effects on plant productivity and water-use efficiency, and heterotrophic respiration will increase as temperature increases^42^; this factor was also not sufficiently considered in this study. The quality of the collected data will also have a strong effect on the accuracy of trait-climate relationships and the training accuracy of GMMs.

N_area_, N_mass_, LMA and LAI were adopted in this paper because they are easy to measure and exhibit high correlations with ecosystem processes. However, they may not be the best candidates for similar studies. The leaf carbon isotope ratio (δ^13^C) of C_3_ plants is inversely related to the drawdown of CO_2_ during photosynthesis^43^, and leaf δ^13^C shows a close relationship with water use efficiency (WUE)^44^. The ratio between the leaf-internal (C_i_) and ambient (C_a_) molar fractions of CO_2_ (C_i_/C_a_) regulates the balance between carbon gain and water loss, which is lower in dry or cold conditions than in wet or hot conditions^26^. Wood density is correlated with mechanical support, water transport and the storage capacity of woody tissues^45^. The maximum carboxylation rate at 25 °C is the key parameter for calculating photosynthesis^46^. These FTs are related to the important role of photosynthesis and reflect the most important functions driving plant establishment, growth, dispersal and competition, which constitute the basic and indispensable structure and function parameters of DGVMs. Future work should consider incorporating these FTs when constructing the next generation of DGVMs. Along with the development of trait-based theories, ecologists have proposed a series of conceptual model frameworks for the next generation of DGVMs based on FTs^13^,^25^,^47^.

Although there are many available results for the prediction of vegetation distributions and ecosystem functions using trait-based methods, there is still a long way to go to integrate these methods into an LSM or EMS. Sakschewski *et al*.^48^ used vegetation individuals with unique key trait combinations to form possible life strategies. These trait combinations varied with climatic factors, which were provided by an LSM or EMS. Another approach is to randomly establish hypothetical growth strategies associated with traits, as in the Jena Diversity-Dynamic Global Vegetation Model (JeDi-DGVM)^13^, and these random traits are affected or filtered using an LSM or EMS. Additionally, a trait-based method should be linked to observations via a model-data fusion approach and should consider the linkage between plant traits and ecosystem functional properties, such as water-use efficiency (WUE), nitrogen-use efficiency (NUE), radiation-use efficiency (RUE), and carbon-use efficiency (CUE), when upscaling to the ecosystem level^49^.

Representing plant species as a set of plant functional traits instead of PFTs provides a new path for analysing ecosystem functions. New trait-based vegetation models can simulate ecosystem functions such as water and carbon cycles better than traditional vegetation models. For this purpose, there are two types of available approaches. The first involves a trait- and individual-based model, such as LPJmL- flexible individual traits (LPJmL-FIT)^48^ or a trait- and individual-based vegetation model (aDGVMs)^25^, which groups individual plants with a number of variable traits. All possible trait combinations represent corresponding growth strategies, with individual plants competing for light and water within the study area. Carbon outputs are calculated by averaging the amount of carbon across all surviving individuals. The second approach is based on the “biomass-ratio” hypothesis, using JeDi-DGVM^13^, and this method links community-aggregated functional traits (i.e., the weight-based mean trait values of all species in a community) and ecosystem functions (i.e., net primary productivity). However, they have been criticized as “not being measurable”^50^ and “not being variable with climate”. Although these methods are still in their early stages, they appear promising, and additional research is needed.

## Materials and Methods

### Selected traits and climate data

In this study, three FTs (leaf mass per area (LMA, g/m^2^), area-based leaf nitrogen (N_area_, g/m^2^), and mass-based leaf nitrogen (N_mass_, %)) and one structural trait of plant communities (leaf area index, LAI) were selected for analysis. In total, we collected 1294 observations (from 1993 to 2013), and each record included at least one of the three FTs (LMA, N_area_ or N_mass_) from the literature published prior to 2014 (Fig. S2, Table S1). We attempted to minimize the uncertainty due to different measurement methods by filtering or correcting data when possible. LAI data were derived from remote sensing products. (More details about trait selection are presented in the Supplementary Information). To remain consistent with global linear trait-climate regressions^38^,^40^,^51^, the mean annual temperature (MAT, °C), mean annual precipitation (MAP, mm) and annual solar radiance (RAD, w/m^2^) were used in this study, which are three of the most important, common climatic variables that cannot be derived from other variables (Fig. S1). Between 1987 and 2013, MAT and MAP were derived from 756 meteorological stations and were interpolated at a 10-km resolution using the software package ANUSPLIN^51^. RAD was calculated using a land-surface-transfer scheme (LSX)^52^,^53^, which was integrated in IBIS DGVM, and temperature, precipitation, relative humidity, wind speed and solar hours were used as input variables.

### An FT- based model: development and simulation strategies

The core of our approach is to build a relationship between climate factors and FTs and to predict vegetation distributions. An earlier conceptual FT-based framework proposed by Douma *et al*.^27^ was modified and improved upon in the present study. Four steps were conducted (Fig. S3): (1) Mathematical models were built to represent the relationships between selected traits and climate variables. (2) FTs and their corresponding observed vegetation types were used in training a GMM, and the trait space was then divided into different sub-spaces in N-dimensional space, belonging to different vegetation types; (3) as inputs of the GMM, the predicted traits under different climatic scenarios were classified into different vegetation types according to the location of the traits in N-dimensional space (expressed as classification probability); (4) as outputs of the GMM, the predicted distribution of vegetation was validated via comparison with natural vegetation maps or observations.

For model training and validation, we randomly divided the data into two parts: half of the data (i.e., 65,657 points) were used for the training of a GMM and the other half were used for model validation. We used different trait combinations (Table 1) to train the GMM and calculate the classification accuracy, after which the optimal combination was applied for a sensitivity analysis (more details of the model evaluations are shown in the Supplementary Information). Finally, the optimal GMM was applied to analyse the sensitivity of vegetation in China under different climate scenarios.

### Classifications with a Gaussian mixture model (GMM)

Gaussian functions are widely applied in statistics for describing normal distributions^54^,^55^. In discriminant analysis, if Gaussian density distributions have been confirmed, the probability associated with each class is easy to obtain. Bensmail and Celeux^54^ applied a Gaussian mixture model (GMM) in discriminant analysis but included only a single Gaussian component for each class. A more flexible alternative is to use multiple Gaussian components in classification^55^,^56^. A GMM is a combination of several individual Gaussian components: a 1-dimensional Gaussian mixture (Equation1) can be represented in 2-dimensional space, and a 2-dimensional Gaussian mixture (Equation 2) can be represented in 3-dimensional Gaussian space (Fig. S4).


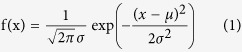



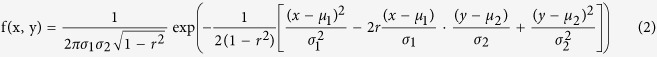


In Eq. 1, f(x) is the occurrence probability function of a 1-dimensional trait belonging to a specific vegetation type, which is also known as a 1-dimensional Gaussian function; x is the independent variable (i.e., trait); and μ is the mean value of the trait sample for a specific vegetation type. σ represents the standard deviation of the sample. In Eq. 2, f(x, y) is the occurrence probability function of 2-dimensional traits belonging to a specific vegetation type, also known as a 2-dimensional Gaussian function. x and y are independent variables (i.e., traits). μ_1_ refers to the mean of the first trait dimension, and μ_2_ refers to the mean of the second dimension. σ_1_ and σ_2_ are the standard deviations of the sampled traits. r^2^ is the correlation coefficient between x and y.

An attractive property of GMMs is that they do not require any arbitrary and potentially restrictive assumptions in the form of probability density functions (PDFs)^55^. GMMs are regarded as an important approach contributing to the construction of the next generation of DGVM based FTs^27^. A GMM can be expressed as in Eq. 3.


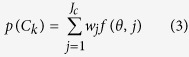


where p(*C*_*k*_) is the Gaussian density of traits belonging to the *C*_*k*_ class; *J*_*c*_ is the number of components; and w_*j*_ represents the components’ weights, such that w_*j*_ > 0, and ∑*w*_*j*_ = 1. *f* (*θ, j*) represents the j^th^ Gaussian component. MCLUST, an R package, was applied in this study^57^.

### Sensitivity analysis

Sensitivity analysis was performed to investigate the response of the predicted vegetation patterns by using the GMM to model the combined effects of changing temperatures and precipitation. Two approaches were adopted in the sensitivity analysis. In the first approach, following the strategy of Wang *et al*.^58^, we designed 56 climate change scenarios that incorporated a uniform increase in temperature up to warming of 5 K. We used 0.5 K intervals from the baseline condition (i.e., the average climate conditions from 1987 to 2013 in China) to 2 K and 1 K intervals, from 2 K to 5 K. Precipitation was both increased and decreased uniformly by up to 30% in 10% increments. The other approach analysed the vegetation distribution under different representative concentration pathways (RCPs); the results are presented in the Supplementary Information (the vegetation sensitivity under future climate change scenarios).

## Additional Information

**How to cite this article**: Yang, Y. *et al*. A novel approach for modelling vegetation distributions and analysing vegetation sensitivity through trait-climate relationships in China. *Sci. Rep.* **6**, 24110; doi: 10.1038/srep24110 (2016).

## Supplementary Material

Supplementary Information

## Figures and Tables

**Table 1:** Properties of the selected trait-climate relationships.

Traits^*^	Observations	Mean	Lower and upper boundaries^#^	Environmental factors^$^	R^2^ adjusted	P-value
Log N_mass_	877	0.33	−0.02, 0.65	−MAT	0.156	<0.01
Log N_area_	2485	0.26	−0.15, 0.65	+RAD, −log (MAP)	0.086	<0.01
Log LMA	3084	1.96	1.48, 2.60	+MAT, −log (MAP)	0.129	<0.01
LAI	1337	0.79	0.00, 3.10	+MAP, −RAD	0.638	<0.01

The traits are N_mass_ (mass-based leaf nitrogen), N_area_ (area-based leaf nitrogen), LMA (leaf mass per area) and LAI (leaf area index). N_mass_, N_area_ and LMA were log10-transformed before analysis. The lower and upper boundaries were based on the 2.5 and 97.5 quantiles, respectively, of all individual observations. The environmental factors are MAT (mean annual temperature), MAP (mean annual precipitation) and RAD (solar radiation). “+” indicates a positive relationship, and “−” indicates a negative relationship in regression analysis.

**Table 2:** Results for selected traits in Gaussian mixture models (GMMs).

ID	Models^*^	Overall accuracy (%)	Kappa coefficient (%)
1	N_mass_, N_area_	65.95	87.51
2	N_mass_, LMA	66.24	87.38
3	N_mass_, LAI	65.37	87.56
4	N_area_, LMA	66.93	87.27
5	N_area_, LAI	57.95	89.16
6	LMA, LAI	65.86	87.57
7	N_mass_, N_area_, LMA	73.46	85.69
8	N_mass_, LMA, LAI	72.82	85.91
9	N_mass_, N_area_, LAI	70.71	86.28
10	N_area_, LMA, LAI	70.80	86.17
11	N_mass_, N_area_, LMA, LAI	68.48	85.85

The traits are N_mass_ (mass-based leaf nitrogen), N_area_ (area-based leaf nitrogen), LMA (leaf mass per area) and LAI (leaf area index). N_mass_, N_area_ and LMA were log10-transformed before analysis.
