# Supplementary material for: A novel approach for modelling vegetation distributions and analysing vegetation sensitivity through trait-climate relationships in China
Source: Sci Rep. 2016 Apr 7;6:24110. doi: 10.1038/srep24110 (PMC4823651; doi:10.1038/srep24110)
Supplement: Supplementary Information [file srep24110-s1.doc]

**SUPPLEMENTARY INFORMATION**

**A novel** **approach for modelling vegetation distributions and analysing vegetation sensitivity through trait-climate relationships in China**

Yanzheng Yang^1, 3^, Qiuan Zhu^1^,Changhui Peng^2, 1^, Han Wang^1^, Wei Xue^1^, Guanghui Lin^3^, Zhongming Wen^1^, Jie Chang^4^, Meng Wang^1^, Guobin Liu^1^ & Shiqing Li^1^

^1^ State Key Laboratory of Soil Erosion and Dryland Farming on the Loess Plateau, Northwest A&F University, Yangling, Shaanxi, China

^2^ Department of Biology Sciences, Institute of Environment Sciences, University of Quebec at Montreal, Montreal, Canada

^3^ Center for Earth System Science, Tsinghua University, Beijing, 100084, China

^4^ College of Life Sciences, Zhejiang University, Hangzhou, 310058, China

Correspondence and requests for materials should be addressed to Q.Z. (qiuan.zhu@gmail.com)

Further details for selected traits and climate data. LMA, N_mass_ and N_area_ are three important FT candidates for improving the accuracy of DGVMs; they have been widely used ^1-5^ and are widely measured in China. LMA measures the leaf dry-mass investment per unit of light-intercepting leaf area and is the inverse of the specific leaf area (SLA). Species with a high LMA exhibit a thick leaf blade, dense tissue or both^6^. LMA is strongly related to the photosynthetic capacity and the potential relative growth rates of plants^7^. N_area_ co-varies with leaf δ^13^C and represents an adaptation to drought and water conservation ^8^. The leaf nitrogen concentration (both mass-based and area-based) is integral to the proteins involved in the photosynthetic machinery, especially Rubisco ^6^. N_mass_ increases with increasing temperature and latitude ^9^. The leaf area index (LAI) as a structural trait of plant communities, is an indicator of canopy cover and annual leaf turnover (only for deciduous trees). LAI directly determines photosynthetic production and exhibits a range of phenological behaviours ^10^. Therefore, not only can we determine the vegetation distribution through trait-climate relationships, but we can also connect FTs to an ecosystem’s structure and functions.

Although GLOPNET^6^ has collected many datasets, it only includes approximately 1% of vascular plant species, and only one site in China is represented ^11^. Consequently, we could not define significant relationships between FTs and climate using only the GLOPNET FT dataset from China. Therefore, we combined 1093 new observed traits collected in China with the 2548 observations available in GLOPNET, thereby supplementing the worldwide trait data and overcoming the effects of insufficient data from Chinese regions. In addition, we used continuous records for the green leaf area from 8-day MODIS LAI data (MOD15A2, 549 periods) ^12^ to derive the LAI-climate relationship; these data were averaged and acquired between March 2000 and February 2012. Given the effect of cloud-contaminated or aerosol-contaminated reflectance, we created a 10-km LAI dataset using the method proposed by Myneni, et al. ^10^, with the same resolution as the climate data. To ensure that the number of observations was of the same order of magnitude as the number of FT observations, and considering that the grid used in the regression reduces the correlation coefficient (R^2^), we randomly selected 1337 evenly distributed sites to analyse the LAI-climate relationship (Fig. S5).

***Evaluation of the results***

In the construction of a GMM, selecting the appropriate number of traits and determining which model is the most accurate are critical steps. Here, the vegetation type with the maximum probability was assigned to each simulating unit. We tested all the models of the selected traits listed in Table 1 and obtained an estimate of the overall accuracy (Eq. S1) and kappa coefficient (Eq. S2) ^13^ after comparing the results with the observed natural vegetation map. For the GMM-based discriminant analysis, the natural vegetation map, together with the predicted trait patterns, was used to train the GMM classifier. Once the GMM density function has been obtained, the probability layers of each vegetation type can be obtained according to Gaussian density functions.


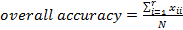
 (S1)


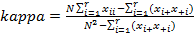
 (S2)

where N represents the number of samples used for evaluating the results of the classification; r is the number of classes; x_i+_ is the total of the i^th^ row in the confusion matrix; and x_+i_ is the total of the i^th^ column. x_ii_ is the value located in the i^th^ row and i^th^ column of the confusion matrix. The threshold value for separating different degrees of agreement according to the kappa coefficient in this study followed Monserud and Leemans ^14^ (Table S2).

***Vegetation sensitivity under future climate change scenarios (RCPs)***

Understanding the vegetation distributions under different future climate change scenarios (RCPs) is important for quantifying the carbon and water cycle budget in the future. BCC-CSM1.1^15^, a climate model developed by the China Climate Centre, was applied to derive three RCPs (typical greenhouse gas concentration trajectories adopted by the IPCC for its Fifth Assessment Report in 2014). The vegetation sensitivity under the three RCPs was analysed, and the results are presented in Fig. S6. As the temperature and irradiation increase from 2025 to 2099 in the three RCPs, the forest vegetation shifts northwest, and subtropical crops expand to cover a larger area. Alpine steppe and temperate desert also expand to cover a larger area compared with the baseline map (see Fig. S6 for more details).

**Figure Captions**

**Fig. S1** Distribution patterns of three environmental variables: mean annual temperature (MAT), mean annual precipitation (MAP) and solar irradiance (RAD). The maps were generated with ArcGIS 10.2, http://www.esri.com/.

**Fig. S2** Trait sampling locations across China used in this study. The size of the symbols represents the number of samples at each site. The maps were generated with ArcGIS 10.2, http://www.esri.com/.

**Fig. S3** Simulation setups used in this study, modified from Douma, et al. ^16^, Van Bodegom, et al. ^3^ and Webb, et al. ^17^. The maps were generated with ArcGIS 10.2, http://www.esri.com/.

**Fig. S4** Example of a 1-dimensional (left) and 2-dimensional (right) GMM. In both cases, the GMM is a linear combination of two pure Gaussian components.

**Fig. S5** Averaged annual MODIS LAI from 2000 to 2012, and the distribution of sampled grids. The maps were generated with ArcGIS 10.2, http://www.esri.com/.

**Fig. S6** Distribution of vegetation for three RCPs in three periods. The legend is the same as in Fig. 2. The maps were generated with ArcGIS 10.2, http://www.esri.com/.

**Table Captions**

**Tab. S1** References for the trait data used in this study.

**Tab. S2** Degree of agreement represented by kappa statistics.


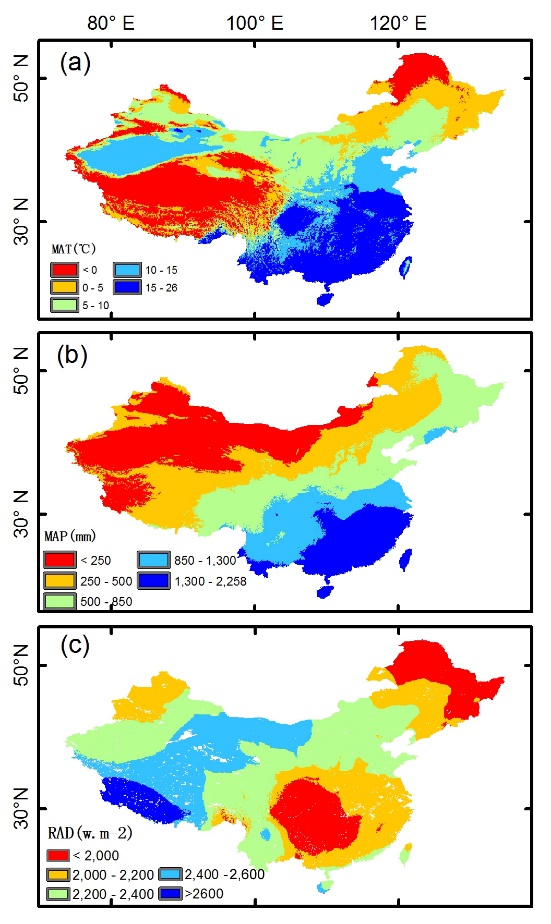


**Fig. S1**


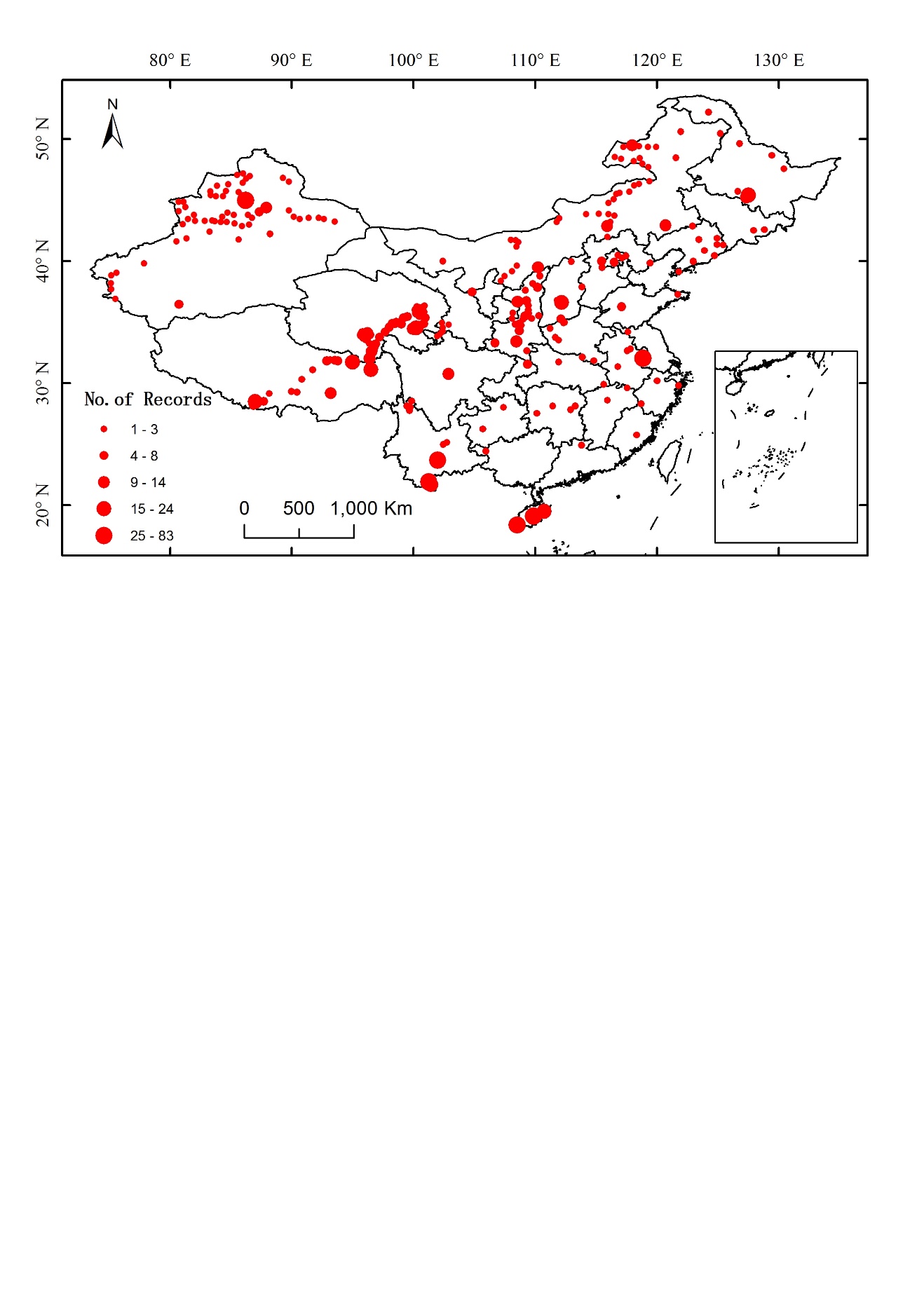


**Fig. S2**

**
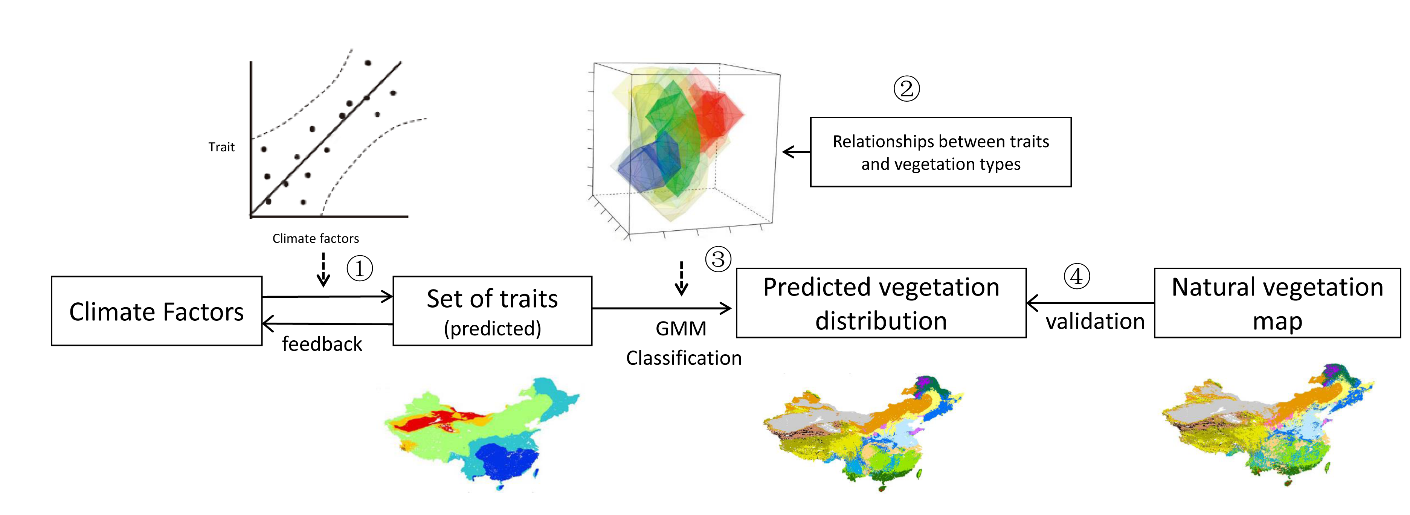
**

**Fig. S3**

**
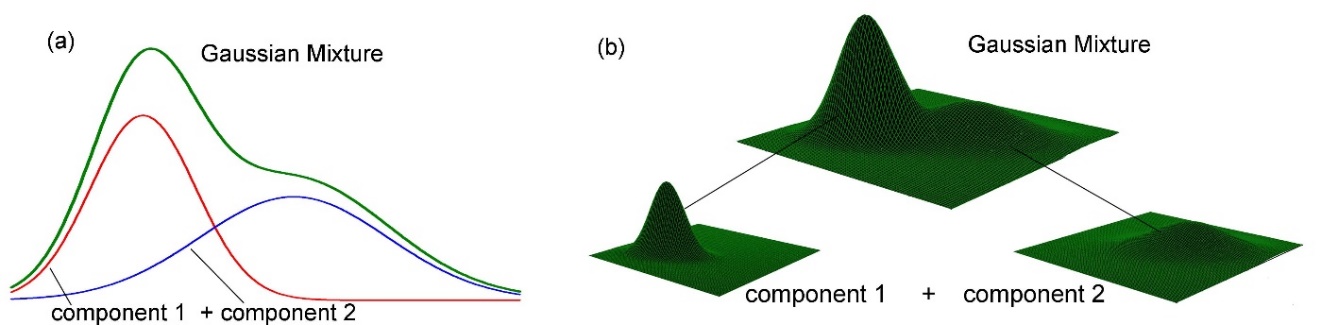
**

**Fig. S4**


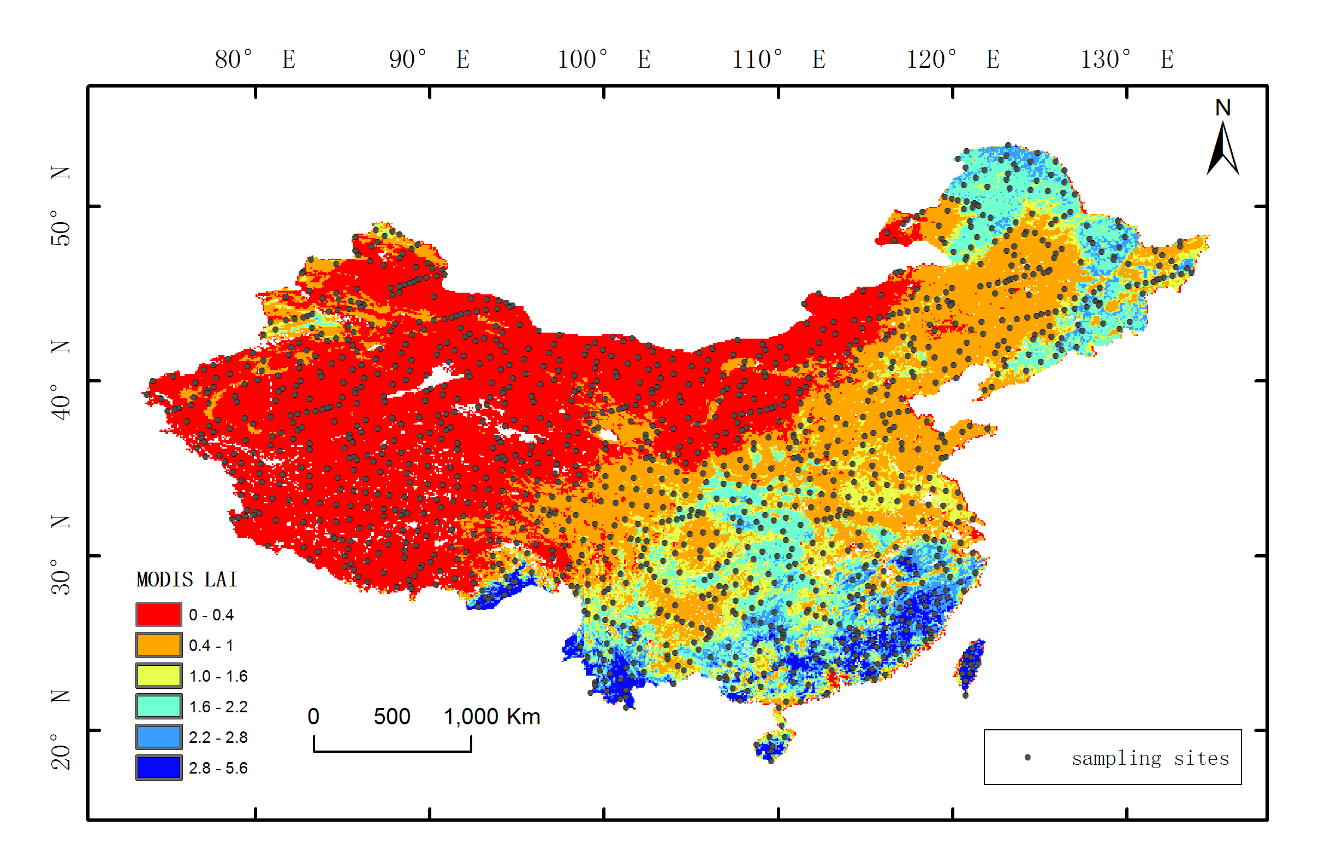


**Fig. S5**

**
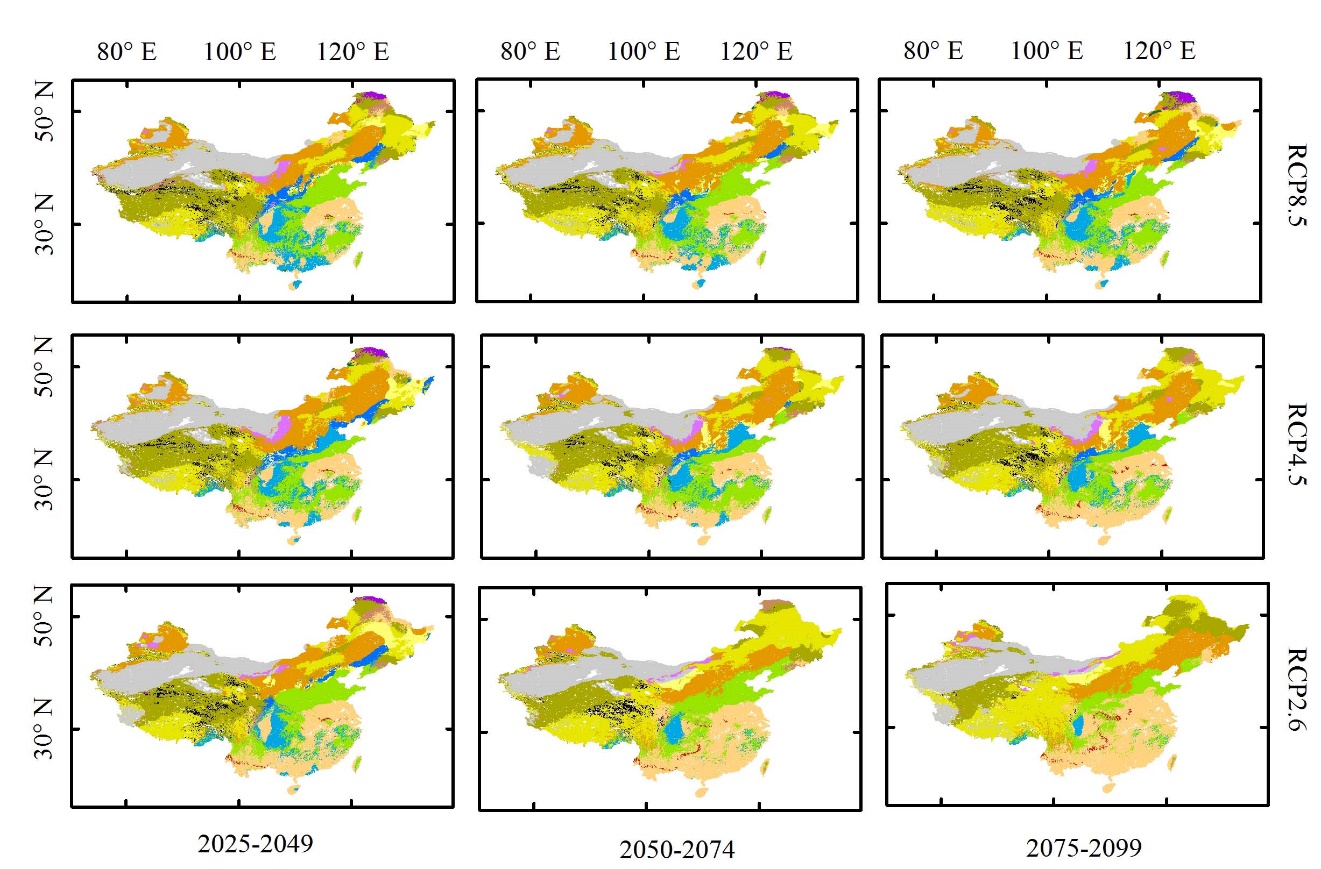
**

**Fig.S6**

**Table S1** References for trait data used in this study.

| ID | LMA | N_area_ | N_mass_ | References |
| --- | --- | --- | --- | --- |
| 1 | √ | √ | √ | Miao, et al. ^18^ * |
| 2 | √ |  |  | Liu, et al. ^19^ * |
| 3 | √ |  |  | Liu, et al. ^20^ * |
| 4 | √ | √ | √ | Li, et al. ^21^* |
| 5 | √ |  |  | Cheng, et al. ^22^ * |
| 6 | √ |  |  | Xu, et al. ^23^ * |
| 7 | √ | √ |  | Zhao, et al. ^24^ * |
| 8 | √ |  |  | Sun, et al. ^25^ |
| 9 |  |  | √ | Wu, et al. ^26^ |
| 10 | √ | √ | √ | Wang, et al. ^27^ |
| 11 | √ | √ | √ | He, et al. ^28^ |
| 12 | √ | √ | √ | Zheng and Shangguan ^29^ |
| 13 | √ |  | √ | Zhang, et al. ^30^ |
| 14 | √ |  | √ | Zhang and Cao ^31^ |
| 15 | √ | √ | √ | Liu, et al. ^32^ |
| 16 | √ | √ | √ | He, et al. ^33^ |
| 17 | √ |  | √ | Cai, et al. ^34^ |
| 18 | √ |  |  | Wu, et al. ^35^ * |
| 19 | √ |  | √ | Song, et al. ^36^ * |
| 20 | √ | √ | √ | Lv, et al. ^37^ * |
| 21 | √ |  |  | Vincent Maire *et al*^38^ |
| 22 | √ | √ | √ | Deng, et al^39^ |
| 23 | √ | √ | √ | Terashima, I *et al*^40^ |

* Papers are written in Chinese.

**Table S2.** Degree of agreement represented by kappa statistics.

| Kappa coefficient | Degree of agreement |
| --- | --- |
| 0-5 | No |
| 5-20 | Very poor |
| 20-40 | Poor |
| 40-55 | Fair |
| 55-70 | Good |
| 70-85 | Very good |
| 85-99 | Excellent |
| 99-100 | Perfect |

**References (Supporting Information)**

1 Pavlick, R., Drewry, D. T., Bohn, K., Reu, B. & Kleidon, A. The Jena Diversity-Dynamic Global Vegetation Model (JeDi-DGVM): a diverse approach to representing terrestrial biogeography and biogeochemistry based on plant functional trade-offs. *Biogeosciences* **10**, 4137-4177, doi:10.5194/bg-10-4137-2013 (2013).

2 Frenette-Dussault, C., Shipley, B., Meziane, D., Hingrat, Y. & Fridley, J. Trait-based climate change predictions of plant community structure in arid steppes. *J. Ecol.* **101**, 484-492, doi:10.1111/1365-2745.12040 (2013).

3 Van Bodegom, P. M. *et al.* Going beyond limitations of plant functional types when predicting global ecosystem-atmosphere fluxes: exploring the merits of traits-based approaches. *Glob. Ecol. Biogeogr.* **21**, 625-636, doi:10.1111/j.1466-8238.2011.00717.x (2012).

4 Westoby, M., Falster, D. S., Moles, A. T., Vesk, P. A. & Wright, I. J. Plant ecological strategies: Some leading dimensions of variation between species. *Annu. Rev. Ecol. Syst.* **33**, 125-159, doi:10.1146/annurev.ecolsys.33.010802.150452 (2002).

5 Verheijen, L. M. *et al.* Impacts of trait variation through observed trait–climate relationships on performance of an Earth system model: a conceptual analysis. *Biogeosciences* **10**, 5497-5515, doi:10.5194/bg-10-5497-2013 (2013).

6 Wright, I. J. *et al.* The worldwide leaf economics spectrum. *Nature* **428**, 821-827 (2004).

7 Reich, P. B., Wright, I. J. & Lusk, C. H. Predicting leaf physiology from simple plant and climate attributes: a global GLOPNET analysis. *Ecol. Appl.* **17**, 1982-1988 (2007).

8 Prentice, I. C., Dong, N., Gleason, S. M., Maire, V. & Wright, I. J. Balancing the costs of carbon gain and water transport: testing a new theoretical framework for plant functional ecology. *Ecol. Lett.* **17**, 82-91, doi:10.1111/ele.12211 (2014).

9 Reich, P. B. & Oleksyn, J. Global patterns of plant leaf N and P in relation to temperature and latitude. *Proc. Natl. Acad. Sci. U. S. A.* **101**, 11001-11006 (2004).

10 Myneni, R. B. *et al.* Large seasonal swings in leaf area of Amazon rainforests. *Proc. Natl. Acad. Sci. U. S. A.* **104**, 4820-4823, doi:10.1073/pnas.0611338104 (2007).

11 Wright, I. J. *et al.* Modulation of leaf economic traits and trait relationships by climate. *Glob. Ecol. Biogeogr.* **14**, 411-421, doi:10.1111/j.1466-822x.2005.00172.x (2005).

12 Fang, H. L., Wei, S. S. & Liang, S. L. Validation of MODIS and CYCLOPES LAI products using global field measurement data. *Remote Sens. Environ.* **119**, 43-54, doi:10.1016/j.rse.2011.12.006 (2012).

13 Ridd, M. K. & Liu, J. J. A comparison of four algorithms for change detection in an urban environment. *Remote Sens. Environ.* **63**, 95-100, doi:10.1016/s0034-4257(97)00112-0 (1998).

14 Monserud, R. A. & Leemans, R. COMPARING GLOBAL VEGETATION MAPS WITH THE KAPPA-STATISTIC. *Ecol. Model.* **62**, 275-293, doi:10.1016/0304-3800(92)90003-w (1992).

15 Xin, X. G. *et al.* How Well does BCC_CSM1.1 Reproduce the 20th Century Climate Change over China? *Terr. Atmos. Ocean. Sci. Lett.* **6**, 21-26 (2013).

16 Douma, J. C. *et al.* Towards a functional basis for predicting vegetation patterns; incorporating plant traits in habitat distribution models. *Ecography* **35**, 294-305 (2012).

17 Webb, C. T., Hoeting, J. A., Ames, G. M., Pyne, M. I. & LeRoy Poff, N. A structured and dynamic framework to advance traits-based theory and prediction in ecology. *Ecol. Lett.* **13**, 267-283, doi:10.1111/j.1461-0248.2010.01444.x (2010).

18 Miao, M. Y., Lv, J. Z. & Bi, R. C. Relationships between Leaf Nitrogen Content and Photosynthetic Characteristics in Different Plant Functional Types. *Bull.* *Bot. Res.* **32**, 425-429 (2012).

19 Liu, J. H., Zeng, D. H. & Don, K. L. Leaf traits and their interrelationships of main plant species in southeast Horqin sandy land. *Chin. J. Ecol.* **25**, 921-925 (2006).

20 Liu, Y. B., Wang, H. F. & Sun, C. Y. Leaf Characters of Four Tree Species at Maoershan Homogeneous Park. *For. Sci. Technol.* **38**, 12-15 (2013).

21 Li, Y. H. *et al.* Comparisons of leaf traits among 17 major plant species in Shazhuyu Sand Control Experimental Station of Qinghai Province. *Acta Ecol. Sin.* **25**, 994-999 (2005).

22 Cheng, J. F., Chen, G. Y. & Shen, Y. G. Leaves characteristics and photosynthetic capacities of various plants in Shennongjia National Nature Reserve. *Ecol. Environ.Sci.* **19**, 165-171 (2010).

23 Xu, Q. H. *et al.* Analysis on Plant Functional Traits of Populus Species in the Tianshan Mountain. *Arid. Zone Res.* **29**, 425-431 (2012).

24 Zhao, X. Y., Wang, C. K. & Huo, H. Variations in photosynthetic capacity and associated factors for Larix gmelinii grom diverse orlgins. *Acta Ecol. Sin.***28**, 3798-3807 (2008).

25 Sun, S. C., Jin, D. M. & Li, R. J. Leaf emergence in relation to leaf traits in temperate woody species in East-Chinese Quercus fabri forests. *Acta Oecol.* **30**, 212-222 (2006).

26 Wu, T. G., Yu, M. K., Geoff Wang, G., Dong, Y. & Cheng, X. R. Leaf nitrogen and phosphorus stoichiometry across forty-two woody species in Southeast China. *Biochem. Syst. Ecol.* **44**, 255-263, doi:10.1016/j.bse.2012.06.002 (2012).

27 Wang, L. M., Mu, M. R., Li, X. F., Lin, P. & Wang, W. Q. Differentiation between true mangroves and mangrove associates based on leaf traits and salt contents*. J. Plant Ecol.* **4**, 292-301 (2011).

28 He, J. S. *et al.* Taxonomic identity, phylogeny, climate and soil fertility as drivers of leaf traits across Chinese grassland biomes. *J. Plant Res.* **123**, 551-561 (2010).

29 Zheng, S. X. & Shangguan, Z. P. Spatial patterns of photosynthetic characteristics and leaf physical traits of plants in the Loess Plateau of China. *Plant Ecol.* **191**, 279-293 (2007).

30 Zhang, J. L., Poorter, L. & Cao, K. F. Productive leaf functional traits of Chinese savanna species. *Plant Ecol.* **213**, 1449-1460 (2012).

31 Zhang, J. L. & Cao, K. F. Stem hydraulics mediates leaf water status, carbon gain, nutrient use efficiencies and plant growth rates across dipterocarp species. *Funct. Ecol.* **23**, 658-667 (2009).

32 Liu, G. F. *et al.* Coordinated variation in leaf and root traits across multiple spatial scales in Chinese semi‐arid and arid ecosystems. *New Phytol.* **188**, 543-553 (2010).

33 He, J. S. *et al.* A test of the generality of leaf trait relationships on the Tibetan Plateau. *New Phytol.* **170**, 835-848, doi:10.1111/j.1469-8137.2006.01704.x (2006).

34 Cai, Z. Q., Schnitzer, S. A. & Bongers, F. Seasonal differences in leaf-level physiology give lianas a competitive advantage over trees in a tropical seasonal forest. *Oecologia* **161**, 25-33 (2009).

35 Wu, L. L., Kang, H. Z., Zhuang, H. L. & Liu, C. J. Variations of Quercus variabilis leaf traits in relation to clmi atic factors at regional scale. *Chin. J. Ecol.* **29**, 2309-2316 (2011).

36 Song, G., Wen, Z. M., Zheng, Y. & Ding, M. Relationships between plant functional traits of Robinia Pseudoacacia and Metrological factors in Loess Plateau, North Shaanxi, China. *Thesis for Master Degree in Northwest A&F University* **20**, 125-130 (2013).

37 Lv, J. Z., Miao, Y. M., Zhang, H. F. & Bi, R. C. Comparisons of Leaf Traits among Different Functional Types of Plant from Huoshan Mountain in the Shanxi Province. *J. Wuhan Bot. Res.* **28**, 460-465 (2010).

38 Maire, V. *et al.* Global effects of soil and climate on leaf photosynthetic traits and rates: Effects of soil and climate on photosynthetic traits. *Glob. Ecol. Biogeogr.* **24**, 706-717 (2015).

39 Deng, X. *et al.* Gas exchange characteristics of the invasive species Mikania micrantha and its indigenous congener M. cordata (Asteraceae) in South China. *Bot. Bul. Acad. Sin. Taipei* **45**, 213-220 (2004).

40 Terashima, I., Masuzawa, T. & Ohba, H. Photosynthetic characteristics of a giant alpine plant, Rheum nobile Hook. f. et Thoms. and of some other alpine species measured at 4300 m, in the Eastern Himalaya, Nepal. *Oecologia* **95**, 194-201 (1993).
